# Diagnostic stability in children and adolescents with bipolar disorder, a nationwide register-based study

**DOI:** 10.1186/s40345-020-0179-3

**Published:** 2020-05-06

**Authors:** Mathilde Frahm Laursen, Rasmus W. Licht, Christoph U. Correll, Tobias Kallehauge, Ann-Eva Christensen, Maria Rodrigo-Domingo, René Ernst Nielsen

**Affiliations:** 1 Psychiatry, Aalborg University Hospital, Aalborg, Denmark; 2 Department of Clinical Medicine, Aalborg University, Aalborg, Denmark; 3 Department of Psychiatry, The Zucker Hillside Hospital, Northwell Health, 75-59 263rd Street, Glen Oaks, New York City, NY USA; 4 Department of Psychiatry and Molecular Medicine, Donald and Barbara Zucker School of Medicine at Hofstra/Northwell, Hempstead, NY USA; 5 Center for Psychiatric Neuroscience, The Feinstein Institute for Medical Research, Manhasset, NY USA; 6 Department of Child and Adolescent Psychiatry, Charité Universitätsmedizin, Berlin, Germany; 7 Unit for Psychiatric Research, Department of Psychiatry, Aalborg University Hospital, Mølleparkvej 10, 9000 Aalborg, Denmark

**Keywords:** Bipolar disorder, Child, Adolescent, Early-onset, Diagnostic stability

## Abstract

**Background:**

Diagnostic stability of bipolar disorder (BD) in children and adolescents, beyond the first contact has been investigated sparsely. The aim of this study was to investigate the diagnostic stability of BD in children and adolescents using over two decades of nationwide register-based data, and to examine factors associated with change from BD to schizophrenia (ICD-10: F20.x), schizoaffective disorder (ICD-10: F25.x) or other primary psychotic disorders (ICD-10 F23.x–24.x and F28.x–29.x).

**Methods:**

Danish register-based data for all incident BD patients diagnosed prior to age 18 years, between January 1st 1995 and December 31st 2014 (N = 519). We graphically illustrated diagnostic change at different follow-up times and studied variables associated with diagnostic change after 3-year follow-up using Poisson regression with robust standard error estimates.

**Results:**

The diagnosis of incident BD was relatively stable. The diagnosis did not change for 93% of those followed for at least 6 months, and remained unchanged for 86% and 73% of those followed at least 3 years and 10 years, respectively. In patients followed for at least 3 years after index BD (N = 478), the risk of diagnostic change was 61% higher in males versus females. The risk of diagnostic change for patients diagnosed during hospitalization was 74% higher compared to patients diagnosed at outpatient clinics/emergency rooms. The risk of diagnostic change for patients abusing substances other than alcohol and cannabis was 173% higher compared to patients not abusing such substances. The risk of diagnostic change for patients previously diagnosed with schizophrenia or related diagnosis was 257% higher compared to patients not having been diagnosed with such diagnosis previously, while the risk of diagnostic change in offspring of parents with schizophrenia or related diagnosis was 126% higher compared to patients who did not have parents diagnosed with such disorders.

**Conclusion:**

Overall, the stability of the BD diagnosis in the Danish nationwide healthcare registers was high. Factors associated with risk of diagnostic change within 3 years of the initial diagnosis were being male, diagnosis given during hospitalization, substance abuse other than alcohol and cannabis, and a prior diagnosis of schizophrenia or related diagnosis in the patient or in their parents.

## Background

When following patients with bipolar disorder (BD) longitudinally, a diagnostic change might indicate either a progression of illness course or a prior lack of diagnostic precision. For example, it has been shown that primary psychotic disorders, i.e., psychotic non-affective disorders, like schizophrenia (SCZ), can have an illness trajectory that develops over a substantial period of time (Salvatore et al. 2012; Bromet et al. 2011).

In the study by Fenning et al. diagnostic stability was defined as the degree to which the original diagnosis is confirmed at follow-up (1994), and in some studies the diagnostic stability has been ascertained by measuring the positive predictive value of the onset-diagnosis compared to the follow-up-diagnosis (Amin et al. 1999). Several studies have estimated the diagnostic stability of adult-onset BD (Fennig et al. 1994; Amin et al. 1999; Marneros et al. 1991; Chen et al. 1998). A register-based study of adults diagnosed with BD during the first psychiatric contact [median age at first contact = 49.0 years (quartiles: 25.1–62.8)] with up to 9 years follow-up, showed that 31.2% had changed diagnosis during follow-up at the 10th contact, with the majority (12.9%) changing to SCZ, schizotypal disorder, and delusional disorders (Kessing 2005). The authors further showed that the stability of the incident BD diagnosis among adults ranged from 85.4% at second contact to 68.8% at the 10th contact, however, information on time related to the contacts was not provided.

In children and adolescents with bipolar disorder, called early-onset BD (EOBD), the diagnostic validity of EOBD has been called into question (Blader and Carlson 2007; Moreno et al. 2007). Despite this debate about EOBD, the stability of single manic episode and BD diagnoses has only been investigated sparsely in youth. Geller et al. (2000) found in 2000 that 85.7% of their cohort of children and adolescents diagnosed with BD as per DSM-IV (American Psychiatric Association 2000) (mean age at baseline was = 10.9 ± 2.7 years) continued to have that diagnosis at the 6-months follow-up, however, the authors did not conduct long-time follow-up. In a register-based study from 2014 by Kessing et al. (2015) the authors showed that 144 children and adolescents aged up to 19 years [median age at first contact = 17.4 years (quartiles: 16.3–18.2)] who were diagnosed with BD at first contact had a diagnostic stability of 76–83% from first to fifth contact, mean follow-up of 1.31 years (quartiles: 0.65–2.48 years). However, in addition to the scarcity of data about the diagnostic stability of BD in patients below age 18 years, detailed and longitudinal information about the change from BD to SCZ, schizoaffective disorder and other primary psychotic disorders are currently missing in this age group.

Thus, the aim of this study was to investigate the degree of diagnostic change in register-defined BD and to explore potential factors for such change in patients below age 18 years.

## Methods

### Design

This was a Danish nationwide register-based cohort study of BD included in the period January 1st, 1995 to December 31st, 2014 followed until April 28th, 2017. Potential factors for a diagnostic change within 3 years of index BD diagnosis were investigated. The contact leading to the first ever BD diagnosis is in the text referred to as the index.

### Sample

The cohort consisted of children and adolescents with a first ever diagnosis of a single hypomanic episode, a single manic episode or BD (ICD-10: F30.x–F31.x) (World Health Organization 1992) during the age of 5–17 years who were registered in the Danish National Patient Register (NPR) (Lynge et al. 2011).

### Registers utilized

The Civil Registration System uses a ten digit unique person identification number (CPR-number) assigned to all Danish citizens upon birth or immigration that allows linking data from different registries (Pedersen 2011). The Civil Registration System contains information on family relationships, and whether the person had emigrated, was missing, or had died at the end of the study period. The Danish Psychiatric Central Research Register (DPCRR) (Mors et al. 2011) contains information on all psychiatric contacts in Denmark from 1969 and onward. The Danish National Patient Register (NPR) (Lynge et al. 2011) contains information from 1977 and onward concerning every contact with any type of hospital in Denmark, including psychiatric hospitals from 1995.

### Measures

#### Outcomes

The main outcome was diagnostic change from BD to any of the following ICD-10 diagnoses: schizophrenia (F20.x), schizoaffective disorder (F25.x) and other primary psychotic disorders (F23.x–24.x and F28.x–29.x) investigated at six different time-points.

The secondary outcome was potential risk factors associated with diagnostic change from BD to schizophrenia (ICD-10: F20.x), schizoaffective disorder (ICD-10: F25.x), or other primary psychotic disorders (ICD-10: F23.x–24.x and F28.x–29.x) for patients followed at least 3 years after index. Patients who received a different diagnosis (i.e. ICD-10: F20.x, 23.x–25.x and 28–29.x) during follow-up are referred to as the *group with diagnostic change,* while those who did not are referred to as the *group with unchanged BD*.

### Procedure

We identified all patients receiving a first ever BD diagnosis and computed their follow-up time after index. We investigated the proportion of remaining patients with a stable BD diagnosis as well as those changing to SCZ, schizoaffective disorder (SA), and other primary psychotic disorders (psychosis not otherwise specified, PNOS) at 6 months, 1 year, 2 years, 3 years, 5 years, and 10 years from index. When relevant, we also present the percentage of patients changing between diagnostic groups (e.g. from PNOS to SCZ). The study sample diminished over time due to administrative censoring.

We described the entire sample at baseline, and investigated potential factors for diagnostic change 3 years after index among the demographic and clinical factors described in the section below. Utilizing anonymized healthcare register-data prohibited us from publishing data in which single patients could be identified, therefore variables or frequency strata containing information on less than four patients and aggregated data based on less than five patients are not shown.

We did not specifically investigate if patients had another diagnostic change back to BD again, after an initial diagnostic change from BD to SCZ, SA and PNOS, nor did we investigate if they had been given other co-morbid psychiatric diagnoses in the study period after the initial diagnostic change, as this was not within the scope of the study.

### Demographic and clinical factors

#### Sex

Sex was defined according to the CPR register (Pedersen 2011).

#### Psychiatric family history

History of psychiatric disorders in the parents was retrieved from the NPR and the DPCRR (Mors et al. 2011) and categorized into: (1) schizophrenia or related diagnoses (ICD-8: 295, 297, 298 excl. 298.0 and 298.1, 299 (World Health Organization 1967) and ICD-10: F20.x–F29.x (World Health Organization 1992), (2) Affective disorders (ICD-8: 296, 298.0, 298.1, 300.4 (World Health Organization 1967) and ICD-10: F30.x–F39.x (World Health Organization 1992), (3) Substance abuse [ICD-8: 291, 294.3, 303, 304 (World Health Organization 1967) and ICD-10: F.10.x–F19.x (World Health Organization 1992)], (4) Other psychiatric disorders (ICD-8: 291 to 315 (excluding the above mentioned) (World Health Organization 1967) and ICD-10: Fxx.x (excluding the above mentioned) (World Health Organization 1992) and [ICD-8: E950–E959, E980–E989 (World Health Organization 1967) and ICD-10: X60–X84, Y10–Y34 (World Health Organization 1992)]. The parents were followed from start of register until 1 year after the EOBD diagnosis of the child.

#### Age at first bipolar-spectrum diagnosis

Age at the index diagnosis was computed based on the date of birth and the date of first single hypomanic episode, single manic episode or first BD (ICD-10 F30.x/F31.x) (World Health Organization 1992), retrieving the diagnosis from the NPR (Lynge et al. 2011). Age at first bipolar-spectrum diagnosis was further dichotomized into EOBD: < 13 vs ≥ 13 years of age.

#### Age at first psychiatric contact

Data on age at first psychiatric contact were retrieved from the DPCRR (Mors et al. 2011) and the NPR (Lynge et al. 2011).

Psychiatric contacts were defined as being registered with a primary diagnosis of any psychiatric disorder [ICD-8: 291–315 (World Health Organization 1967) and ICD-10: Fxx.x (World Health Organization 1992)], or intentional self-harm acts [ICD-8: E950–E959, E980–E989 (World Health Organization 1967) and ICD-10: X60–X84, Y10–Y34 (World Health Organization 1992)].

#### Latest psychiatric diagnosis prior to BD diagnosis

Data on psychiatric contacts were retrieved from the NPR (Lynge et al. 2011) and the DPCRR (World Health Organization 1967) and categorized into: (1) schizophrenia or related diagnoses (ICD-8: 295, 297, 298 excl. 298.0 and 298.1, 299 (World Health Organization 1967) and ICD-10: F20.x–F29.x (World Health Organization 1992), (2) Affective disorders [ICD-8: 296, 298.0, 298.1, 300.4 (World Health Organization 1967) and ICD-10: F30.x–F39.x (World Health Organization 1992), (3) Substance abuse (ICD-8: 291, 294.3, 303, 304 (World Health Organization 1967)] and ICD-10: F.10.x–F19.x (World Health Organization 1992), (4) Other psychiatric disorders (ICD-8: 291 to 315 (excluding the above mentioned) (World Health Organization 1967) and ICD-10: Fxx.x (excluding the above mentioned) (World Health Organization 1992) and [ICD-8: E950–E959, E980–E989 (World Health Organization 1967) and ICD-10: X60–X84, Y10–Y34 (World Health Organization 1992)].

#### Substance abuse

Information on substance abuse was retrieved from the DPCRR (Mors et al. 2011) and the NPR (Lynge et al. 2011) and coded into two separate dichotomous variables with the possible levels of yes and no. The first variable was substance abuse of any type before or at time of first manic episode or first BD diagnosis, including alcohol (ICD-8: 291 and 303 (World Health Organization 1967) and ICD-10: F10.x) (World Health Organization 1992), cannabis (ICD-8 304.5 (World Health Organization 1967) and ICD-10: F12.x) (World Health Organization 1992), or substances other than alcohol or cannabis [ICD-8: 294.3, 304 excl. 304.5 (World Health Organization 1967) and ICD-10: F11.x, F13.x–F19.x] (World Health Organization 1992). The second variable was abuse of substances other than alcohol or cannabis (ICD-8: 294.3, 304 excl. 304.5 (World Health Organization 1967) and ICD-10: F11.x, F13.x–F19.x) (World Health Organization 1992) before or at time of first manic episode or first BD diagnosis.

#### Diagnostic setting

Data on first ever BD diagnosis were retrieved from the NPR (Lynge et al. 2011) and sub-divided into: (1) diagnosed during hospitalization or (2) diagnosed at outpatient clinic or in the emergency room (ER).

#### Period of diagnosis

The year of diagnosis was retrieved from the NPR (Lynge et al. 2011) and classified into two periods: (1) First BD diagnosis between January 1st 1995 and December 31st 2004, or (2) First ever BD diagnosis between January 1st 2005 and December 31st 2014, since the number of patients newly diagnosed with BD has increased substantially since 2005.

#### Psychotic BD diagnosis

When the first ever BD diagnosis was registered as psychotic BD (ICD-10: F30.2, F31.2 and F31.5) (World Health Organization 1992), dichotomized into yes/no.

### Statistical analysis

Demographic and clinical data were described using total numbers and percentages or means and standard deviations, where appropriate.

The percentage of patients with a stable BD diagnosis and those changing to other diagnoses was illustrated graphically at different time-points. A sensitivity analysis was conducted excluding patients diagnosed with SCZ prior to first BD diagnosis.

Poisson regressions with robust standard errors were used to calculate the relative risk (Zou 2004) for diagnostic change between the groups of sex, abuse of alcohol and cannabis or other substances, diagnostic setting, psychotic BD diagnosis, year of diagnosis, latest psychiatric diagnosis prior to BD, psychiatric family history, respectively, as well as for an increase of one in the continuous variables age at first ever BD diagnosis, and age at first psychiatric contact, respectively. Results with p < 0.05 were considered statistically significant.

Statistical analyses were performed with Stata 15 (StataCorp. 2017. Stata Statistical Software: Release 15. College Station, TX: StataCorp LLC).

## Results

Baseline demographic and clinical characteristics for all children and adolescents with a first ever BD diagnosis between 1995 and 2014 (N = 519) as well as for those followed at least 3 years (N = 478) are present in Table 1. A lag of 1.5 years between the first psychiatric contact and first ever BD diagnosis was shown as well as only 7% of the sample being below age 13 years at time of first BD diagnosis (Table 1).

**Table 1:** Characteristics of bipolar disorder (BD) in children and adolescents between 1995 and 2014

Variables	Total population included		Total population with 3-year follow-up	
	N	%	N	%
Children and adolescents with BD	519	100	478	92
Sex (females)	299	58	274	57
Family history of psychiatric disorders	162	32	–^a^	–^a^
Family history of bipolar disorder	57	11	–^a^	–^a^
Substance abuse total	53	10	48	10
Substance abuse, other than alcohol and cannabis	25	5	23	5
Diagnostic setting				
Inpatient	201	39	188	39
Outpatient	318	61	290	61
Psychotic BD at index	92	18	90	19
Index BD prior to 2005	131	25	130	27
Index BD < 13 years	36	7	32	7
Latest psychiatric diagnosis prior to BD				
Schizophrenia or related diagnosis	93	18	86	18
Affective disorders	130	25	117	24
Substance abuse	40	8	37	8
Other psychiatric disorders	286	55	263	55
Information about parents				
Schizophrenia or related diagnosis	44	9	–^a^	–^a^
Affective disorders	111	22	–^a^	–^a^
Substance abuse	77	15	–^a^	–^a^
Other psychiatric disorders	9	2	–^a^	–^a^

Variables	Mean	SD	Mean	SD
Age at first ever BD	15.9	2.1	15.9	2.1
Age at first psychiatric contact	14.4	3.6	14.3	3.6

^a^Numbers could not be reported in order to comply with the data protection rules set by Statistics Denmark in order to avoid allowing identification of individual patients in cells containing ≤ 5 patients

### Diagnostic stability

**Fig. 1:**
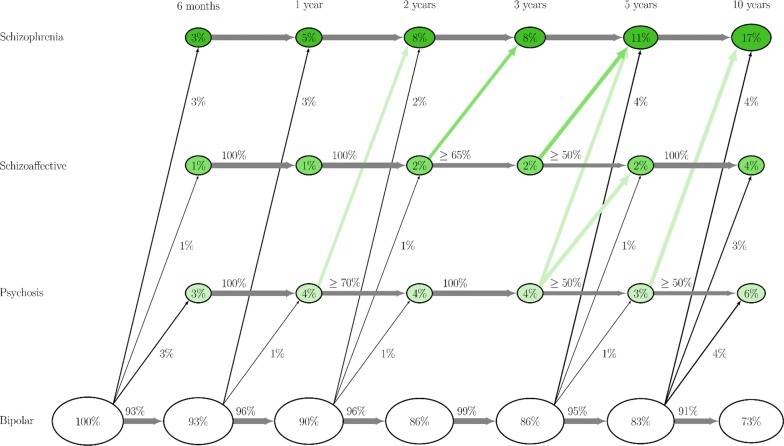
Percentages of patients who changed diagnosis and patients continuing to have the diagnosis latest given to them. Percentages are computed using the number of patients available in the sample at each time point (N = 519 at baseline, N = 478 at 3 years follow-up, N = 169 at 10 years follow-up). The sum of the percentages in the ovals for each time point is 100%, as is the sum of the arrow percentages (both horizontal and rightwards), except for rounding errors. Arrows representing less than four patients are depicted as green arrows without percentages if originating from Psychosis (PNOS) or Schizoaffective (SA) groups, or not represented at all if originating from Bipolar (BD) group

Altogether, 519 patients received a first ever BD diagnosis during the study period. Within 6 months of the index diagnosis, 3% had changed diagnosis to SCZ, 1% to SA, and 3% to PNOS. The percentages that changed diagnosis at 1, 2, 3, and 5 years were similar, but were slightly higher for those followed at least 10 years (see Fig. 1).

Figure 1 shows transitions from BD to the specific diagnoses of SCZ, SA or PNOS after 6 months, 1, 2, 3, 5 and 10 years. Results show that only a minor proportion changed from PNOS or SA to SCZ over time, and almost none changed diagnosis from PNOS to SA. Furthermore, diagnostic change from BD to SA or PNOS was relatively rare. Diagnostic change from BD to PNOS was at most 6%, being 4% or less at all time-points for conversions from BD to SA. Most of the patients who changed diagnosis from BD did so to SCZ, and after 10 years of follow-up, 17% of the available sample (n = 169) had changed diagnosis to SCZ. Still, the majority (73%) of the patients with initial BD diagnoses did not change diagnosis even 10 years after the index BD diagnosis.

Our sensitivity analyses showed that excluding patients diagnosed with SCZ prior to the first ever BD diagnosis did not change percentages presented in Fig. 1 substantially (data not shown).

Altogether, 478 patients (92.1% of the original sample) were followed for at least 3 years. Of those patients, 86% continued to have an BD diagnosis whereas 14% had changed diagnosis to SCZ, SA or PNOS (Fig. 1). Forty-one patients were followed for less than < 3 years and had a mean ± SD follow-up time of 2.39 ± 0.74 years. Of those, 11 children and adolescents changed diagnosis from BD to SCZ, SA or PNOS.

### Factors associated with diagnostic change

Table 2 presents the number and percentages for the examined factors in the group with an unchanged BD diagnosis and in the group with a diagnostic change at 3 years follow-up, as well as the RR of a diagnostic change.

**Table 2:** Bipolar disorder (BD) in children and adolescents with and without diagnostic change after 3 years of follow-up (N = 478)

	Group with unchanged BD (N = 412)	Group with diagnostic change (N = 66)	RR (95% CI)	*p* value
	N (%)	N (%)		
Sex				0.038
Female	244 (89.1%)	30 (10.9%)		
Male	168 (82.4%)	36 (17.6%)	1.61 (1.03; 2.53)	
Family history of psychiatric disorder				0.310
No	–^a^ (87%)	–^a^ (13%)		
Yes	–^a^ (84%)	–^a^ (16%)	1.27 (0.80; 2.03)	
Family history of BD				0.629
No	–^a^ (86%)	–^a^ (14%)		
Yes	–^a^ (88%)	–^a^ (12%)	0.82 (0.37; 1.18)	
Substance abuse (total)				0.286
No	373 (86.7%)	57 (13.3%)		
Yes	39 (81.3%)	9 (18.8%)	1.41 (0.75; 2.68)	
Substance abuse of other than alcohol and cannabis				0.001
No	397 (87.3%)	58 (12.7%)		
Yes	15 (65.2%)	8 (34.8%)	2.73 (1.48; 5.02)	
Diagnostic setting				0.015
Outpatient or ER	259 (89.3%)	31 (10.7%)		
Inpatient	153 (81.4%)	35 (18.6%)	1.74 (1.11; 2.73)	
Psychotic BD at index				0.055
No	340 (87.6%)	48 (12.4%)		
Yes	72 (80.0%)	18 (20.0%)	1.62 (0.99; 2.64)	
Index BD prior to 2005				0.988
No	300 (86.2%)	48 (13.8%)		
Yes	112 (86.2%)	18 (13.8%)	1.00 (0.61; 1.66)	
Index BD < 13 years				
No	384 (86.1%)	62 (13.9%)		
Yes	> 27 (–%)^1^	< 5 (–%)^1^	–^a^	–^a^
Latest psychiatric diagnosis prior to BD				
Schizophrenia or related diagnosis				< 0.0001
No	355 (90.6%)	37 (9.4%)		
Yes	57 (66.3%)	29 (33.7%)	3.57 (2.33; 5.47)	
Affective disorders				0.568
No	313 (86.7%)	48 (13.3%)		
Yes	99 (84.6%)	18 (15.4%)	1.16 (0.70; 1.91)	
Substance abuse				0.655
No	381 (86.4%)	60 (13.6%)		
Yes	31 (83.8%)	6 (16.2%)	1.19 (0.55; 2.57)	
Other psychiatric disorders				0.134
No	191 (88.8%)	24 (11.2%)		
Yes	221 (84.0%)	42 (16.0%)	1.43 (0.90; 2.29)	
Information about parents (N = 473)				
Schizophrenia or related diagnosis				0.003
No	–^a^ (88%)	–^a^ (12%)		
Yes	–^a^ (72%)	–^a^ (28%)	2.26 (1.32; 3.90)	
Affective disorders				0.680
No	–^a^ (87%)	–^a^ (13%)		
Yes	–^a^ (85%)	–^a^ (15%)	1.12 (0.66; 1.91)	
Substance abuse				0.928
No	–^a^ (86%)	–^a^ (14%)		
Yes	–^a^ (86%)	–^a^ (14%)	1.03 (0.55; 1.92)	
Other psychiatric disorders				0.438
No	–^a^ (86%)	–^a^ (14%)		
Yes	–^a^ (> 55%)	–^a^ (< 45%)	1.64 (0.47; 5.68)	

	Mean (SD)	Mean (SD)	RR (95% CI)	p-value
Age at first ever BD diagnosis (N = 478)	15.9 (2.1)	16.0 (2.1)	1.01 (0.91; 1.13)	0.857
Age at first psychiatric contact (N = 478)	14.4 (3.5)	14.0 (4.3)	0.98 (0.92; 1.04)	0.465

^a^The exact numbers could not be reported in order to comply with the data protection rules set by Statistics Denmark in order to avoid allowing identification of individual patients in cells containing ≤ 5 patients

In patients followed for at least 3 years after index BD (N = 478), males had a RR = 1.61 (95% CI (1.03; 2.53), p = 0.038) for a diagnostic change compared to females. Patients diagnosed during hospitalization had a RR = 1.74 (95% CI 1.11; 2.73), p = 0.015) for a diagnostic change compared to patients diagnosed at outpatient clinics/ER. Patients abusing substances other than alcohol and cannabis had a RR = 2.73 (95% CI (1.48; 5.02) p = 0.001) for a diagnostic change compared to patients not abusing such substances. Patients previously diagnosed with SCZ or related disorder had a RR = 3.57 (95% CI (2.33; 5.47), p  < 0.001) for a changing diagnosis, compared to patients who had not been diagnosed with SCZ previously. Patients whose parents had previously been diagnosed with a SCZ or a related diagnosis had a RR = 2.26 (95% CI (1.32; 3.90) p = 0.003) for a diagnostic change compared to patients without parents with SCZ or a related diagnosis (Table 2).

## Discussion

Our investigation of the long-term diagnostic stability of BD according to the Danish registers showed that among patients with follow-up information 93% of the diagnoses were stable after 6 months, 90% after 1 year, 86% after 2 and 3 years, 83% after 5 years, and 73% after 10 years, respectively.

The stability of BD in the Danish registers was investigated previously by Kessing et al. (2015), who in the period from 1994 to 2012 in a cohort of 354 children and adolescents (age 0–19 years) found that, at their second psychiatric contact, 79.6% out of 98 patients continued to have a BD diagnosis and at fifth contact 77.3% out of 22 patients with follow-up information still had the BD diagnosis. The results by Kessing et al. (2015) resemble the findings in the current study although the time intervals between visits are not reported by Kessing et al. (2015) and we only know that the total follow-up time was longer than 2.5 years for only 25% of the sample. Therefore, a direct comparison between the results from our study with the results of the second, third and fourth visits in the study by Kessing et al. (2015) were not possible, although both studies present supporting evidence of high diagnostic stability of the BD diagnosis in the Danish registers.

Furthermore, Kessing et al. found that 3.1% at the second contact, increasing to 4.6% at the fifth contact, had changed diagnostically to SCZ or a related diagnosis (ICD-10 F20.x–29.x). However, that study did not investigate the proportions who changed from BD to SCZ specifically. Therefore, the findings in the current study demonstrating that 3% had changed diagnosis to SCZ after 6 months and 17% after 10 years cannot be directly compared to findings by Kessing et al. (2015).

The risk of diagnostic change was significantly higher for (1) males than females, (2) in-patients compared to outpatients and patients diagnosed in the ER, (3) patients who were using substances other than alcohol and cannabis, (4) patients diagnosed with a SCZ or related diagnosis before index BD, and (5) patients with parents who had been previously diagnosed with a SCZ or related diagnosis. Diagnostic change might have been a consequence of illness progression with patients fulfilling the diagnostic criteria for ICD-10 BD at time of first ever BD diagnosis, and with a further progression in the psychopathology later on, leading to the diagnostic shift to PNOS, SA or SCZ seen at later contacts. The findings could also be a results of patients being misdiagnosed with BD in the first place due to an overlap of symptoms (Murray et al. 2004). However, notably, psychotic BD was not a significant factor for a diagnostic shift to SCZ or a related disorder. Nevertheless, affective disturbances have been shown to be a part of the SCZ prodrome (Cornblatt et al. 2003) and, as such, symptoms of a SCZ prodrome could potentially be misdiagnosed as BD. Why males had a higher risk for changing diagnosis than females at the 3 year follow-up is unclear, but could be ascribed to the fact that males develop SCZ at an earlier age than females (Usall et al. 2002). Indeed, males might have fulfilled diagnostic criteria for BD at baseline, but due to illness progression, a substantial proportion would have fulfilled diagnostic criteria for a SCZ or related diagnosis 3 years after first BD diagnosis.

Lastly, BD can be more difficult to diagnose in children and adolescents than in adults, as reflected in two similar studies using different age groups by Kessing et al. (2005, 2015) who found that only 40.7% of children and adolescents got a diagnosis of mania or BD at first in- or outpatient contact in contrast to 56.2% of adults. In the current study the vast majority of the sample was diagnosed with an incident BD registry diagnosis between 13 and 18 years of age, making the study mostly comparable to other studies investigating BD in adolescents. However, future studies should focus of identifying symptoms that overlap, characteristics with the highest specificity for a diagnostically stable BD, or a diagnostically stable SCZ disorder diagnosis, and predictors of diagnostic change from BD to SCZ.

## Strengths and limitations

The results of this study need to be interpreted in light of several limitations. First, we investigated only false positives, but not false negatives, i.e., to what degree patients are misdiagnosed and there is a substantial delay between the actual illness onset and the diagnosis of BD in children and adolescents. Second, we only investigated if patients with BD had diagnostic change to a SCZ, SA or PNOS, but we did not investigate all diagnoses given subsequently. It is possible to receive another BD diagnosis after a SCZ, SA or PNOS diagnosis, but we considered this as unlikely and therefore we did not investigate this issue. In a study by Laursen et al. (2019), using the Danish registers, it was found that only 2.3% of children and adolescents below age 18 years changed diagnosis from SCZ to BD during the study period (1995–2014). Third, we did not investigate comorbidities. After receiving a BD diagnosis, patients might later on be referred to psychiatric care primarily due to an ICD-10 F4.x diagnosis, e.g. anxiety (Faedda et al. 2014; Duffy et al. 2016), however, such conditions are frequently comorbid with BD, for which reason, BD would still be present. Fourth, we included single hypomanic episode and single manic episode as part of BD since we believed that a single hypomanic episode or a single manic episode will most likely be followed by another mood episode (Kessing 2005; Goodwin 2002). Fifth, there is a possibility of underreporting of the true incidence of BD because a proportion of BD might be misdiagnosed with e.g. major depression (Lish et al. 1994; Hirschfeld et al. 2003). Furthermore, the registry reflects clinical practice and is influenced by referral to hospital-based psychiatry as well as diagnostic culture. Sixth, we were unable to ascertain the true sensitivity as well as specificity of the BD diagnosis in the register because there is no information about the false and true negatives. Finally, results may not fully generalize to other countries, regions and health care systems, which is why similar studies are needed in other areas of the world. In the current study only few changed diagnostic group to PNOS and SA, thus, it was not possible to analyze whether factors associated with increased risk of diagnostic change would differ in those groups, compared to what was shown in Table 2. Lastly, in the current dataset we do not have SES data available and as such we were unable to investigate the effects thereof on diagnostic change.

Despite these limitations, this study has also several strengths. First, we used population-based nationwide registers and, since public healthcare in Denmark is free of charge, we included all patients diagnosed with BD without exclusion based on socioeconomic status or geographical location, as is the case in some insurance database healthcare studies. Second, the study had minimal loss to follow-up owing to the mandatory reporting of all data on in-patients and out-patients to the Danish health care registers. Third, we have been able to follow the majority of patients for at least 3 years after index and a quite large proportion for up to 10 years, which to our knowledge is the longest follow-up period investigating the diagnostic stability of BD.

## Conclusion

The diagnosis of the BD in the Danish registers is relatively stable. The risk for diagnostic change from BD to SCZ, SA, or PNOS 3 years after first ever BD diagnosis are higher for males, inpatients, patients who had a SCZ or related diagnosis prior to first ever BD diagnosis, patients using substances other than alcohol and cannabis, and for patients whose parents had received a SCZ or related diagnosis. These risk factors should be considered and used in the screening and diagnostic practice as well as when following patients with BD longitudinally.

## Data Availability

The datasets used and analysed during the current study are only available after permission obtained from the Danish Health Data Authorities and Statistics Denmark.

## Diagnoses-related

EOBD: Early-onset bipolar disorder
BD: Bipolar disorder
SA: Schizoaffective disorder
SCZ: Schizophrenia
PNOS: Psychosis not otherwise specified (other primary psychotic disorders)

## Regarding statistics

SD: Standard deviations
RR: Relative risk

## Regarding the registers used

DPCRR: Danish Psychiatric Central Research Register
NPR: National Patient Register
CPR: Central Person Register (Civil registration System)

## General

ER: Emergency room
